# Controlled Bulk Properties of Composite Polymeric Solutions for Extensive Structural Order of Honeycomb Polysulfone Membranes

**DOI:** 10.3390/membranes6020027

**Published:** 2016-05-16

**Authors:** Annarosa Gugliuzza, Maria Luisa Perrotta, Enrico Drioli

**Affiliations:** 1 Research Institute on Membrane Technology-National Research Council (CNR-ITM), Via Pietro Bucci 17C, Rende (CS) 87036, Italy; ml.perrotta@itm.cnr.it (M.L.P.); e.drioli@itm.cnr.it (E.D.); 2 Department of Chemical Engineering and Materials, University of Calabria, Via Pietro Bucci, 33C, Rende (CS) 87036, Italy; 3 WCU Energy Engineering Department, College of Engineering, Hanyang University, Seoul OK763, Korea

**Keywords:** breath figure membranes, extensively ordered textures, water self-assembly

## Abstract

This work provides additional insights into the identification of operating conditions necessary to overcome a current limitation to the scale-up of the breath figure method, which is regarded as an outstanding manufacturing approach for structurally ordered porous films. The major restriction concerns, indeed, uncontrolled touching droplets at the boundary. Herein, the bulk of polymeric solutions are properly managed to generate honeycomb membranes with a long-range structurally ordered texture. Water uptake and dynamics are explored as chemical environments are changed with the intent to modify the hydrophilic/hydrophobic balance and local water floatation. In this context, a model surfactant such as the polyoxyethylene sorbitan monolaurate is used in combination with alcohols at different chain length extents and a traditional polymer such as the polyethersufone. Changes in the interfacial tension and kinematic viscosity taking place in the bulk of composite solutions are explored and examined in relation to competitive droplet nucleation and growth rate. As a result, extensive structurally ordered honeycomb textures are obtained with the rising content of the surfactant while a broad range of well-sized pores is targeted as a function of the hydrophilic-hydrophobic balance and viscosity of the composite polymeric mixture. The experimental findings confirm the consistency of the approach and are expected to give propulsion to the commercially production of breath figures films shortly.

## 1. Introduction

Well-defined porous polymeric architectures are in a great demand for use in advanced devices, including membranes [1,2,3] and sensors [4,5,6]. There is a deep-seated awareness that desired properties can be decided and programmed or more simply controlled at the macroscopic level when chemistry and tight placement of each single component are properly directed on multiple length scales, including nanometers and micrometers. This necessarily implies an extensive structural order over the entire surface area that must be managed. As an example, extensive structural order is highly desired for the implementation of water membrane processes, including membrane distillation. In this case, narrow pore distribution together with pore size and shape decide extensiveness and stability of the interfacial area, which are necessary for an efficient process with a long operational time [1]. Also, the increasing demand to move from passive to active sensors requires the use of platforms wherein sensing and transport functions can be allocated and associated according to fine structure-property relationships [5].

Several manufacturing methodologies have been proposed for the fabrication of highly defined textures [6,7,8,9,10], but often overly expensive materials/production processes, the use of pollutant materials, as well as the loss of controlled texture at long range have resulted in being too restrictive for related scale-up. Among the most breakthrough strategies, there is one inspired to by the natural condensation of water droplets from humid air on cold surfaces, which is well known as breath figure self-assembly [11]. The approach is based on a bottom-up strategy and exploits the ability of water droplets to self-assemble in semi-crystalline lattices under dragging Marangoni forces. The propagation of the droplet assembly through the polymeric solution leaves air bubble arrays, where each single pore can be regarded as the result of the imprinting action of a droplet. Such a technique has important advantages over conventional approaches, including the use of largely available and nontoxic template and a reduced amount of solvent as well as the requirement of only one fabrication step. All this makes it attractive for the greener and time-saving design of well-architectured interfaces from several polymers. Indeed, this kind of honeycomb structure has been successfully proven to work as a highly breathable membrane when equipping membrane distillation plants [1,12] as well as porous platforms for the build-up of high-quality charge transfer pathways for humidity detection and regulation [13]. However, the commercial development of advanced devices based on materials with this long-range texture type necessarily requires a massive production. A current limitation to the breath figure method is, indeed, the difficulty to control the organization of droplets into ordered and modular structures over the entire surface area exposed to the humid air, especially when polymers with a low ability to stabilize droplets are used. In addition, the technique is rather sensitive to changes, which can take place in the surrounding environment, and the perfect control of the external conditions is not trivial at a larger scale.

Despite the literature referring to several models of honeycomb structures [14,15,16], the formation of highly ordered multiscale polymeric textures still remains a hard struggle. On the other hand, thermodynamics, kinetics, and entropy factors, which regulate the materials assembly, are generally somewhat complex and unclear [17,18,19,20,21].

Currently, there is a still considerable lack of knowledge about the forces dominating the degree of structural order on the scale, while the scale-up of defined structures realistically needs to use practical and efficient means for achieving desired features at longer range.

Also, there is the necessity to demonstrate this technique as a practical route for the preparation of honeycomb membranes with open regular pores from a large number of polymers, including those with traditionally poor affinity to water and scarce ability to stabilize floating droplets at longer range.

In this context, this study provides additional insights into the identification of synergic interactions to accomplish the entrapment and motion of water droplets through a continuous hydrophobic phase, thus preventing the formation of locally disordered regions at the boundary of touching droplet islands and yielding full order throughout the surface. A further advantage is the possibility to make polymeric architectures with a modular pore diameter by directing nucleation and growth rate events through the manipulation of kinetic and thermodynamic factors, including viscosity and surface free tension. Herein, a common polyethersulfone (PES) with low affinity to water is chosen as a polymer type due to its excellent thermal stability, outstanding toughness and suitability to come in contact with food and water. Also, a commercial nonionic surfactant such as polyoxyethylene (20) sorbitan monolaurate (Tween20) is chosen as a model surfactant and is combined with alcohols with different lengths and bulks of the chain. The aim is to generate different chemical microenvironments and study the behavior of floating water droplets during self-assembly as the hydrophilic/hydrophobic balance in the solution is changed. Fine driving forces, including encapsulation and reciprocal affinity of touching systems, are tuned at the interfacial level and are concerned with the organizations of droplet lattices achieved over the entire surface area exposed to the humid atmosphere. As a result, a very high degree of order is obtained through honeycomb film textures with well-shaped and well-sized pores when fruitful interactions are established at the water-casting solution interface. The major result is the suppression of disordered and confused regions as a suitable hydrophilic/hydrophobic balance is provided to the polymeric solution. In this case, it is demonstrated how the combination of PES/surfactant/alcohols enables one to move from traditional self-assembly to assisted self-assembly, taking the advantage of packing pores with modulated size in extensively controlled PES honeycomb geometries where local disorders are entirely suppressed.

These experimental achievements can be regarded as the result of well-identified tools, which represent, in turn, a solid precondition to the scale-up of reliable interfaces with structural features which are in accordance with requirements of regular and uniform pore size, high interfacial area and defined and reproducible volumetric space.

## 2. Experimental Section

### Materials and Methods

Polyethersulfone (PES, Radel A100NT, Solevey Solexis, Alpharetta, GA, USA) was dissolved in dicloromethane (DCM, 99.5%, Carlo Erba Reagents, Milan, Italy) at 2.0 wt %. Polyoxyethylene (20) sorbitan monolaurate (Tween20, *M_w_* = 1227.54, Sigma-Aldrich SRL, Milan, Italy) and various alcohols with different chain length ((CH_2_)*_n_*_= 2–4_OH, water content < 0.02%, degree of purity of 99.5%, Carlo Erba Reagents) were added at different ratios in the polymer solution, resulting in a concentration of 10^−5^–10^−3^ M for the surfactant and 12 wt % for alcohols. The clear dopes were placed in clean and dried stainless steel supports having an area of around 4 cm^2^ located inside a pre-equilibrated box at 20 °C and under a partial pressure of water vapor of 17.54 mmHg until the films were formed. The viscosity of the various polymer dopes was measured at 20 °C by a capillary rheometer (*c* = 0.00243 cst/s). Dynamic Light Scattering (DLS) measurements were carried out to evaluate changes in the aggregation state by using a 90 plus Particle Size Analyzer (Malvern Instruments Ltd., Worcestershire, UK). The surface free tensions of the solutions were estimated according to the pendant drop method by using a micro-syringe with an automatic dispenser and a digital camera to capture images (CAM 200-KSV Instrument Ltd., Helsinki, Finland), while the parameters of solubility were calculated according to the following equations [22]:

(1) $$e_{coh}=\;{\left(\frac{\gamma_{s}}{0.75}\right)}^{\frac{3}{2}},\;\delta =\;{\left(e_{coh}\right)}^{1/2}$$

where *e_coh_* (10^6^ J/m^3^) is the energy cohesion density and δ (10^3^ J^1/2^/m^3/2^) the solubility parameters. Interfacial tensions between water and dichloromethane solutions containing polymer at 2.0 wt % and Tween20, within a range of concentration from 0 to 10^−3^ M, were also measured by pendant drop method at 20 °C. Each solution was poured in the quartz glass cuvette, and then a water drop was injected into the solution by using a syringe connected to an automatically liquid volume dispenser. The interfacial tensions were measured at 0 and 60 s after water injection. The average interfacial tension values were calculated from five measurement results with a standard deviation that decreases from ±0.23 to ±0.13 with rising surfactant content into the mixture [23].

Membrane morphology was investigated by scanning electron microscopy (SEM; Quanta FENG 200, FEI Company, Milan, Italy). Pore size and pore distribution were estimated from SEM images by using the Image J software (version 1.37, Softonic Internacional S.A., Barcelona, Spain). The pore size had a log-normal distribution for all membranes and was expressed as the probability density function [24].

The cumulative distribution function was obtained for each single membrane by plotting the median rank on the ordinate *versus* the ascending pore size on the abscissa and was a straight line on log-normal probability paper with correlation coefficients R^2^ higher than 0.90.

## 3. Results and Discussion

**Figure 1:**
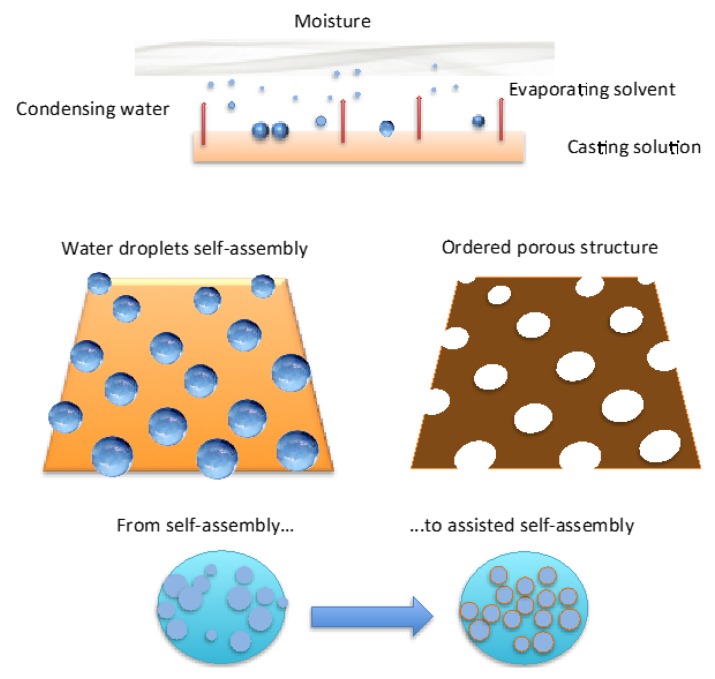
Representative scheme of formation of ordered porous membranes according to breath figure method.

The breath figure is regarded as a greener and time-saving manufacturing approach for ordered porous honeycomb structures (Figure 1).

**Figure 2:**
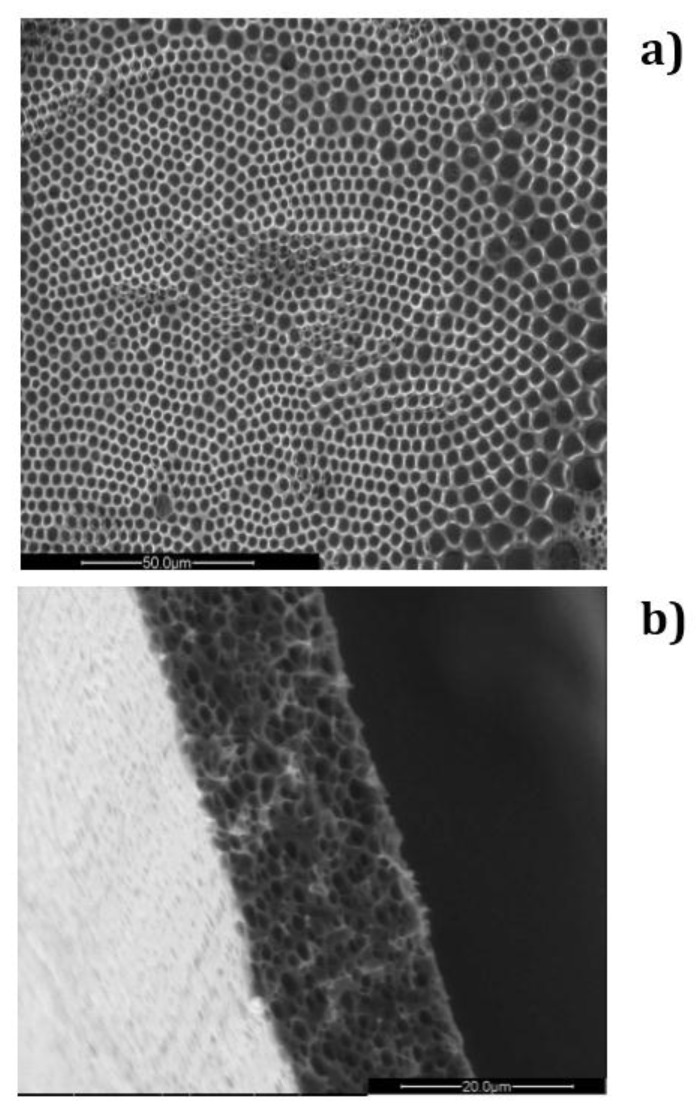
SEM micrographs related to a honeycomb membrane prepared from a PSU/DCM/2-propanol solution: (**a**) top surface and (**b**) cross-section of the membrane.

As mentioned, the target is to use water droplets as natural pore builders, thus yielding three-dimensionally-defined polymeric architectures (Figure 2). However, a major limitation to commercially producing large-scale films is the lack of control on the structural order at long-range through the surface area of the films realized.

Briefly, when liquid films come in contact with humid air, water condensation on the liquid surface is induced for cooling effects due to solvent evaporation (Figure 1). After nucleation, floating water droplets form more or less extended domains apart from each other. These domains are dragged by thermo-capillary forces that would direct the self-assembly to the area exposed to the humid air. At the same time the polymer has the ability to envelope the droplets when touching the solution and form a semisolid protective layer around each one. However, as the polymer layer is too weak and/or harsh and virulent collisions occur at the margin of the droplet rafts, coalescence phenomena take place shaping more or less extensive irregular and/or inhomogeneous regions upon the starkness of the impact (Figure 2).

With this concern, the purpose of this work is to identify one way to reduce and prevent local disorder through controlled kinematic and thermodynamic forces in order to give new inputs to the production of this kind of membrane on a larger scale, especially for polymers exhibiting low ability to stabilize water droplets during flotation through the solution. The possibility to modulate the pore size within packed honeycomb geometries is explored as well.

For this reason, compounds, by virtue of their capability to rearrange and interact with different chemical environments, have been chosen as models to study. A common surfactant such as Tween20 has been used alone and in combination with alcohols, including ethanol, n-propanol, 2-propanol, and n-butanol, in order to increase the local kinematic viscosity of PES solutions, but also to generate chemical microenvironments favorable for hosting and stabilizing water droplets during condensation and flotation events. PES has been demonstrated to be poor in stabilizing droplets in the absence of surfactant, while extensively regular honeycomb textures have been obtained when surface-active molecules have been used to strengthen cooperative interfacial forces [25].

### 3.1. Influence of the Surfactant on the Surface Structural Order

**Figure 3:**
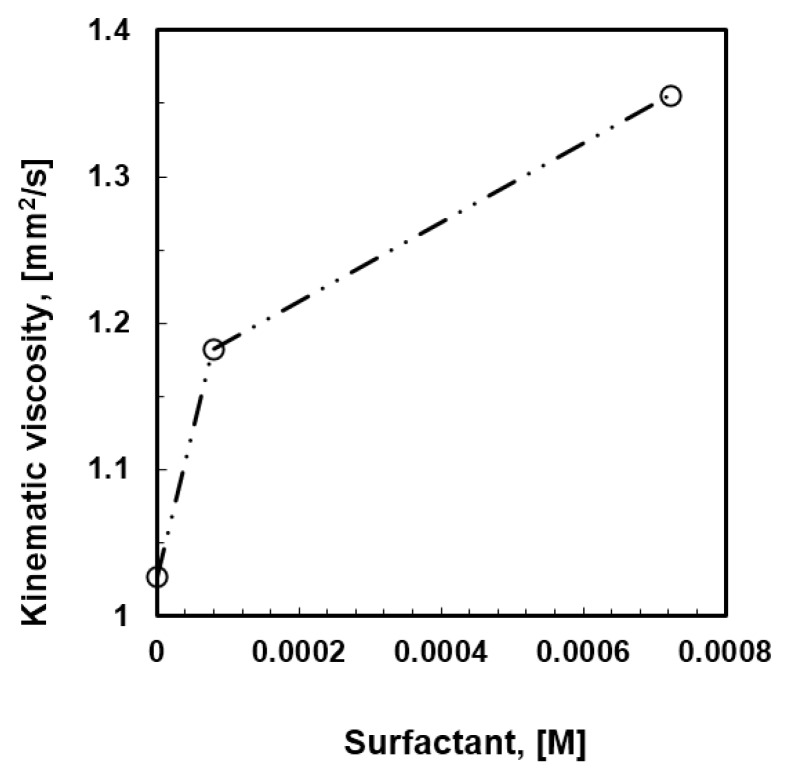
Effects of the surfactant loading on the kinematic viscosity of the PSU/DCM/2-propanol (12 wt %) solutions.

Initially, mixtures of PSU in DCM containing 2-propanol at 12 wt % and Tween20 at different content (10^−5^–10^−3^ M) were prepared. The behavior of the solutions with increasing content of surfactant has been examined when coming in contact with humid air, while kinetics and thermodynamic aspects have been analyzed as well. As the concentration of the surfactant increases, a local increase in gelation comes through the solution, resulting in an increased kinematic viscosity (Figure 3).

**Figure 4:**
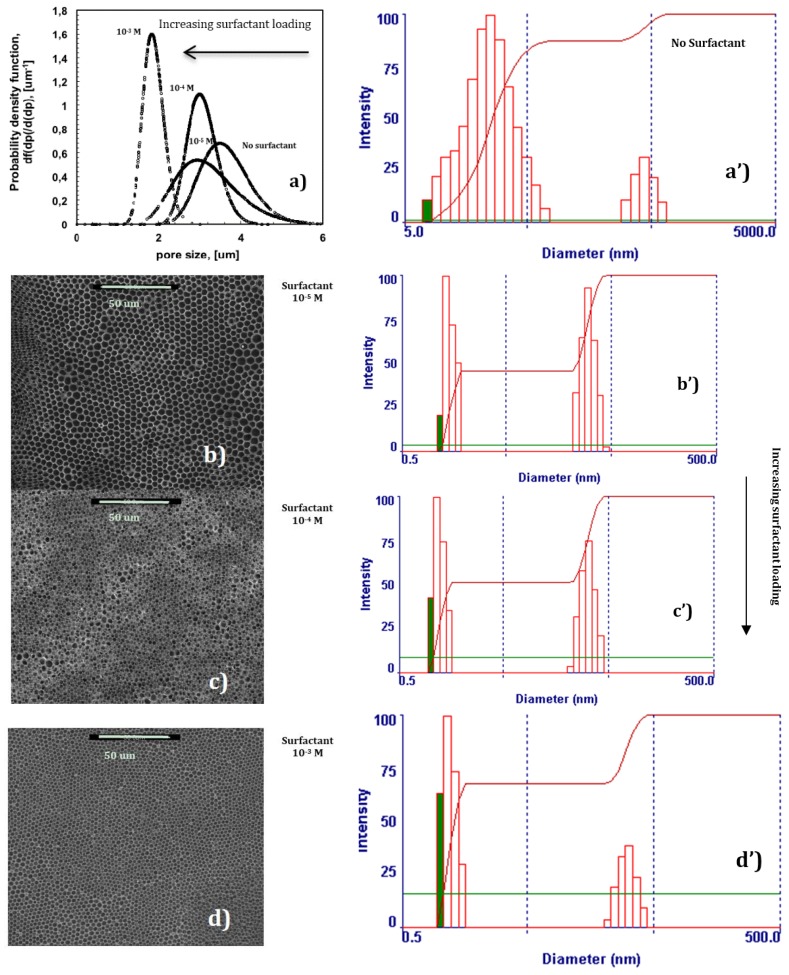
Changes in the pore size and distribution with rising content of surfactant (**a**); Effects of surfactant/alcohol complex on membrane morphology (SEM micrographics (**b**–**d**) and formation of aggregates in precursor polymeric solutions (Dynamic Light Scattering (**a’**–**d’**)).

In the absence of surfactant, 2-propanol is unable to provide the assistance necessary to fully prevent local disorder for the polymer PES, as shown in Figure 2a. Undesired broadness in the pore distribution takes place due to the coexistence of different porous domains, thus resulting in an average pore size of approximately 3.6 um (Figure 4a).

Instead, a progressive addition of Tween20 to the solution leads to the gradual control of the dynamics of the water droplets, making the collisions softer. SEM micrographs show how larger and geometrically confused air bubbles progressively make space for more defined lattices as the content of the surfactant increases in the mixture (Figure 4b–d). This suggests a slowing down of the droplets during motion, which avoids coalescence or uncontrolled growth rate at the margin of the single lattices. As a result, an enhanced degree of the order of the texture is obtained (Figure 4a). In this context, the surfactant assists the polymer in the stabilization of droplets during self-assembly, producing additional fluid viscosity at the droplet-solution-droplet interface. The reduced kinetics prevents the nearest soft particles from merging in bigger bubbles, limiting or, at best, suppressing local disorder at the boundary of the droplet islands. Dynamic Light Scattering (DLS) experiments yield further indications about a cooperative interaction between the surfactant and polymer (Figure 4a’–d’). In the absence of surfactant, two distinct populations of aggregates can be appreciated in solution within a size range of 5–5000 nm, the first one covering a broader range of heterogeneous assemblies (Figure 4a’). The gradual addition of the surfactant in solution significantly reduces the broadness of two populations, decreasing the aggregates’ size by one order of magnitude, and leads to a gradual diminution of the second population in favor of the smallest one (Figure 4b’–d’). Because of the complexity and heterogeneity of the mixtures, establishing the shape and type of the aggregates is not easy at this stage; however, it is undoubtedly due to the relationship between the increasing uniformity of aggregates in solution and the major order of the final texture, which can be regarded as the result of a cooperative action of the various components dispersed in the mixture. It is also relevant to observe how a mono-dispersive pore distribution matches with a gradual reduction of the pore size as the surfactant rises in content (Figure 4).

**Figure 5:**
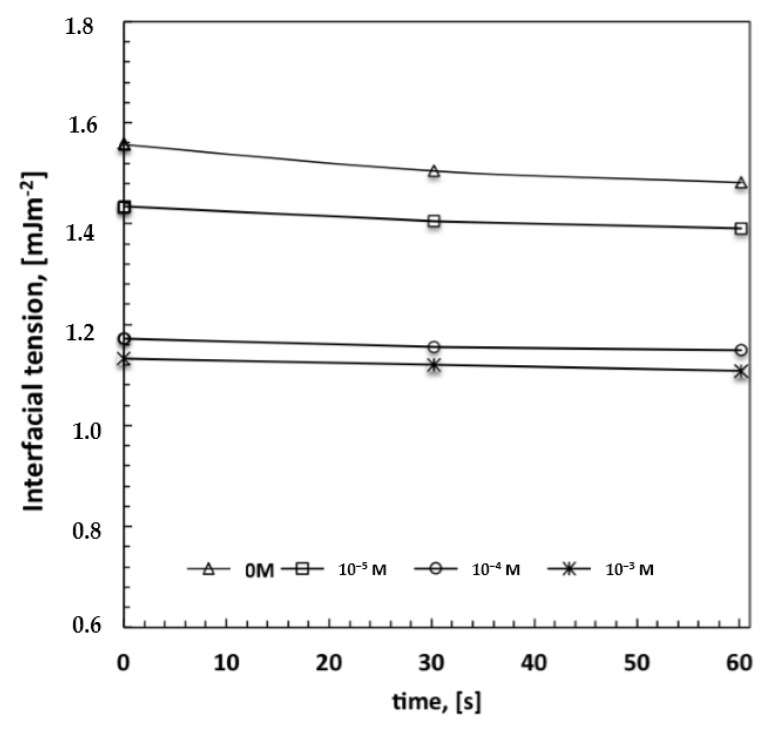
Estimation of interfacial tension values between a water droplet and polymeric solution containing increasing amount of surfactant.

Given that the radius of the droplets is time-dependent and proportional to *R* < *t*^1/3^ in the beginning, and to *R* < *t* in the end [15], very narrow pore size distributions with the formation of a smaller pore size can be regarded as the result of a massive nucleation and a reduced droplet growth rate. There seems to be quick droplet saturation over the liquid surface in contact with moisture, while the boundary of each single droplet island becomes indiscernible at nearly the highest loading of surfactant (Figure 4d). In this regard, the imprinting action of the droplets is exhausted when the lattice is formed over the entire surface area of the solution touching the moisture. However, this can also be considered a reasonable consequence of a very fast and assisted moisture uptake. Indeed, an increase in the overall surface tension—a value of 28.30 mJ/m^2^ against 26.50 mJ/m^2^ estimated for the pure solvent—has been measured for solutions at the highest amount of surfactant. This would suggest that a large number of polar head groups is directed outward from the surface and is prepared to interact with condensing droplets. In order to confirm the ability of the surfactant to interact favorably with the water droplets, interfacial tensions have been measured at the interface established between a single water droplet and the surrounding polymeric solution with increasing content of Tween20. The droplets have been automatically injected into the solution by using a syringe and the surface tension value has been measured according to the pendant drop method. Indeed, the decrease in the interfacial tension values with rising concentration of the surfactant confirms a tendency of the system to reach a minimum of energy (Figure 5). This means that when the surfactant dissolved in the polymeric solution comes in contact with water droplets, the related polar heads establish attractive hydrophilic interactions at the interface while the hydrophobic tails are pointed towards the rest of the nonpolar solution, thus causing a decrease in the overall interfacial tension. These experimental findings are in full agreement with those found by Kojima *et al.* [23] about the ability of amphiphilic copolymers to establish hydrophilic interfacial forces with water droplets during the formation of honeycomb patterns. Herein, the ability of the surfactant to enhance the process of stabilization of the droplets becomes much stronger at higher concentrations, resulting in a higher uniformity of the structural order as well as in a gradual reduction of the pore size (Figure 3a–d). This means that the larger availability of the surfactant leads to quicker water uptake and stabilization over the entire surface area of the solution exposed to humid air.

On this basis, there is a clear indication about the necessity to adjust the hydrophilic/hydrophobic balance in solution in order to move water droplets from self-assembly to assisted self-assembly, especially when using polymers with poor ability to rearrange themselves and interact at the interface of local different microenvironments.

It is relevant to observe how the hydrophilic/hydrophobic balance becomes somewhat marked at higher concentrations of surfactant, which is greater than that indicated as a necessary to reach the critical micelle concentration in a binary aqueous solution (CMC, 10^−2^ mM) [26]. In this respect, it must be stressed that the working chemical environment is rather different from the aqueous one, the mixture being nonpolar and containing four different components. This makes it difficult to unequivocally identify the aggregation state of the surfactant, especially in the presence of additional amphiphilic compounds such as alcohols, which allow intermolecular interactions to establish within hydrophilic and hydrophobic domains, causing important changes in CMC as well [27]. Nevertheless, it is unquestionable that the increase in the concentration of surfactant causes a major number of monomers in proximity to the surface and in the bulk; these monomers could aggregate but also continue to migrate freely towards the surface, making polar heads promptly more available to interact with water and assist the dispersion of the aqueous phase in the continuous oil phase, as clearly confirmed by decreasing interfacial tension values (Figure 5).

The result is a quicker formation of lattices from a larger number of smaller, stabilized water droplets, which leave an open pore size uniformly distributed over the entire surface area of the film. This clearly implies a predominance of droplet nucleation over the related growth rate.

### 3.2. Influence of the Alcohol Chain Length on the Pore Size

**Figure 6:**
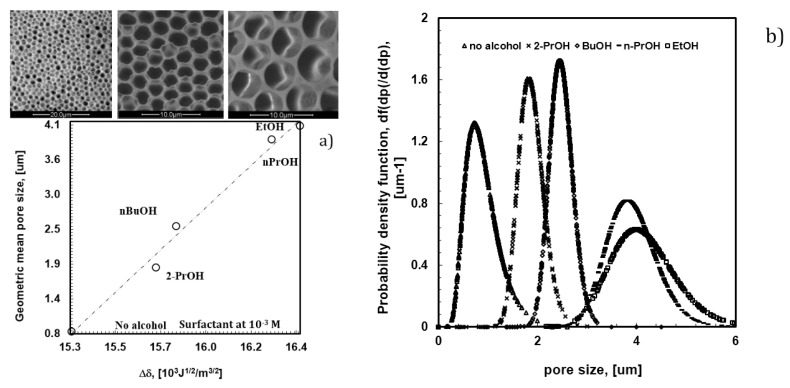
Variation of pore size (**a**) and pore distribution (**b**) as a function of the different length and bulky chain of alcohols contained in PSU/DMC solutions with surfactant at 10^−4^ M.

The pore size can be regarded as the result of a different balance between droplet nucleation and the growth rate steps. A massive droplet nucleation is, in fact, expected to lead to a very fast coverage of the surface with a formation of smaller pores, whereas a lengthy rearrangement extends the growth rate, yielding bigger air bubbles. In this respect, the chemistry of the polymeric mixture has been further changed with the purpose to direct the time-scale of nucleation and the growth rate, respectively. Alcohols with different structure (CH_2_)*_n_*_= 2–4_ containing the OH end group have been added to the mixture, keeping the concentration of the surfactant constant at 10^−4^ M, in order to examine the effects of changing hydrophobic/hydrophilic balance on the pore formation. As shown in Figure 6, the addition of alcohols to the polymeric mixture brings about a marked effect on the value of the overall surface free tension. The latter tends to increase with the length and bulky chain of the alcohol, while the pore size decreases from 4.0 to 0.8 um through the overall film surface.

It is instructive to stress that the increase in the surface free tension affects the scale of affinity, causing inevitably higher values of the solubility parameter (δ) of the solution. The latter is a thermodynamic indicator of the attractive or repulsive interactions established between two systems coming in contact [28]. The difference between the solubility parameters (Δδ, 10^3^J^1/2^/m^3/2^) of two media yields a clear indication about the level of affinity; thus, small differences indicate a great affinity, whereas large differences suggest a poor attraction. Concerning the systems investigated in this study, smaller differences have been estimated between the solubility parameters of water (72.86 mJ/m^2^ at 20 °C) and the composite solutions when the lipophilic component of the alcohol overcomes the hydrophilic one. In this case, a smaller pore size is obtained (Figure 5a). Differently, a larger pore size is measured for films prepared from solutions containing alcohols with higher polar character (Figure 6a).

Undeniably, alcohols with shorter tails exhibit increased polarity and have a higher ability to interact with the polar heads of the surfactant, thus reducing their availability towards water droplets. On the contrary, alcohols with longer and bulky chains exhibit more amphiphilic character, taking their dissolution closer to the hydrophobic regions of the surfactant, where dispersive cooperative intermolecular interactions are better established. This implies a larger availability of the polar heads to face water droplets. As a result, a quicker nucleation of droplets with formation of smaller pore size is obtained when polar heads of the surfactant are more available. This can be envisaged as a direct consequence of a higher hydrophobic molecular interaction established into the bulk of the polymeric solution.

In this regard, it is also relevant to observe that such an availability of the head polar groups at the solution surface-air interface becomes much higher in the absence of alcohols. This suggests a decisive role of the alcohol in the rearrangement of the surfactant at the water-solution interface. Indeed, the difference between the solubility parameters of water and the casting solution is somewhat low in absence of alcohol (Figure 6a). In this case, intermolecular interactions between alcohols and surfactant fail necessarily and a larger number of free monomers in solution orient the polar part outward, yielding major availability to interact with floating droplets. Under these conditions, a larger number of small droplets are formed and stabilized over the entire surface area in contact with humidity. The consequence is a massive nucleation, which leads to highly ordered textures with pores of 0.8 um (Figure 6b).

**Figure 7:**
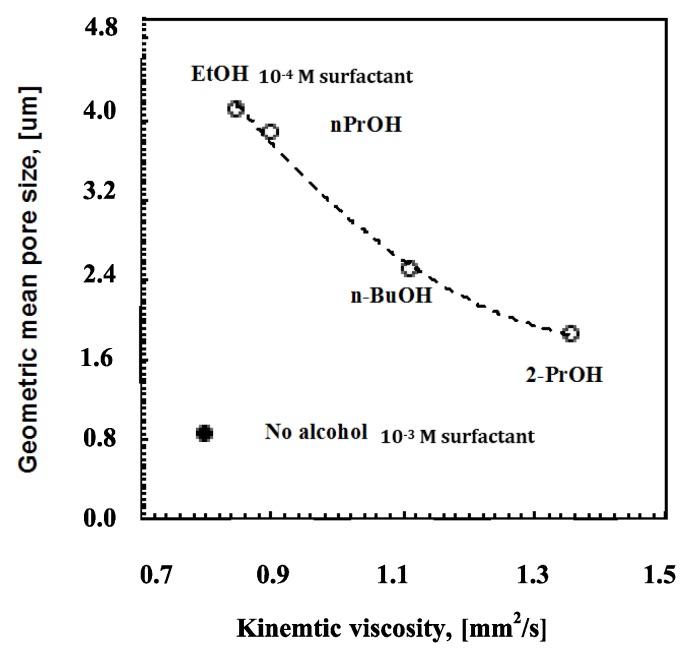
Geometric pore size *vs.* kinematic viscosity for solutions (o) containing surfactant at 10^−4^ M and alcohols with different length and bulky chains.

Again, it is also relevant to examine the incidence of the alcohol length chain on the kinematic viscosity. Figure 7 shows how the rising molecular weight together with the bulky structure of the alcohol causes an effective increase in the solution viscosity (Figure 6).

This is expected to further enhance the capability of encapsulation of the polymeric solution, thereby reducing the risk of coalescence and harsh collisions.

The viscosity factor becomes, however, non-influential in the absence of alcohols; the rate of moisture uptake and coverage for the entire surface area rather than speedy droplet flotation seems to decide the smaller pore arrangement in ordered textures. Comparing a solution of PSU/DMC containing surfactant at 10^−4^ M with ethanol at 12 wt % and a solution of PSU/DMC containing surfactant at 10^−3^ M without alcohol, similar values of kinematic viscosity can be appreciated (Figure 7); however, a significant reduction of around 80% is observed for the pore size as the polymeric solution contains surfactant alone. This suggests that different mechanisms can take place during droplet self-assembly. The surfactant favors moisture uptake and a quicker nucleation rate, whereas the alcohol competes with water in the establishment of intermolecular interactions with the surfactant, reducing the related degree of freedom with the effect of extending the growth rate of the floating droplets depending on the intrinsic polar character of the mixture.

On this basis, the surfactant seems to have a decisive and predominant role in the uptake and stabilization of floating water droplets, whereas the alcohol affects the time scale, resulting in a modular pore size.

It is unquestionable that changes in the hydrophilic-hydrophobic balance cause a competition between droplet nucleation and growth rate steps. Using other classes of amphiphilic compounds, changes in this balance are expected to further modify the time scale with consequences on the final texture of the polymeric porous film [13].

The intent of this work is to demonstrate the necessity to move from traditional self-assembly to assisted self-assembly approaches as a precondition to make the technique scalable, thus preserving structural order and yielding uniformly modulated pore size at longer range. Of course, the precondition for a successful scale-up has to pass through the adjustment of thermodynamic and kinetic parameters and, consequently, the manipulation of bulk properties of the solutions used, enabling one to contrast undesired effects due to low ability of the polymer to stabilize the droplets but also to the frequent sensitivity of the droplet assembly to little changes in the external environment.

## 4. Conclusions

Assisted self-assembly of droplets in a honeycomb pattern is herein discussed. Composite polymeric solutions have been prepared and exposed to moisture according to the breath figure approach. Condensing water droplets have been directed to generate uniform and well-shaped pores in polymeric architectures over the entire surface area of the films. In order to get water droplets in extensively ordered honeycomb textures, changes in the hydrophilic-hydrophobic balance of the solution have been induced using polyoxyethylene (20) sorbitan monolaurate as a traditional surfactant. The latter has been also combined with alcohols with increasing length and bulky chains in order to obtain pore size within a broad range of microns. Depending on the local microenvironment generated, droplet nucleation and growth rate steps have been competitively promoted due to controllable forces established at the water-solution interface. Kinematic viscosity and water affinity have been discerned in relation to the degree of order and the size of the pores packed in honeycomb geometry. Changes in the interfacial tension have been concerned with the ability of amphiphilic components of the solution to stabilize water droplets through the hydrophobic fluid. The role of the hydrophilic-hydrophobic balance in the competition between the nucleation and growth rate has been discussed. As a result, it has been demonstrated that the manipulation of the polymeric solution makes a better control of the water droplet dynamics at longer range possible, leading to open pores uniformly packed in orderly, extensive honeycomb textures. This is expected to give a great boost to the scale-up of this fabrication process, which has the potential to meet the increasing demand to commercially produce large-scale ordered porous films at low cost.

## Abbreviations

The following abbreviations are used in this manuscript:

CMC	critical micelle concentration
DCM	dicloromethane
DLS	Dynamic Light Scattering
*e_coh_*	energy cohesion density
PSU	polysulphone
SEM	scanning electron microscopy
Tween20	polyoxyethylene (20) sorbitan monolaurate
γ	surface free tension
δ	solubility parameter
Δδ	difference of solubility parameters
